# The genetic component of Brugada syndrome

**DOI:** 10.3389/fphys.2013.00179

**Published:** 2013-07-15

**Authors:** Morten W. Nielsen, Anders G. Holst, Søren-Peter Olesen, Morten S. Olesen

**Affiliations:** ^1^The Danish National Research Foundation Centre for Cardiac ArrhythmiaCopenhagen, Denmark; ^2^Department of Cardiology, Laboratory for Molecular Cardiology, The Heart Centre, Rigshospitalet, University of CopenhagenCopenhagen, Denmark

**Keywords:** Brugada syndrome, genetics, Exome Sequencing Project, mutation, treatment

## Abstract

Brugada syndrome (BrS) is a clinical entity first described in 1992. BrS is characterized by ST-segment elevations in the right precordial leads and susceptibility to ventricular arrhythmias and sudden cardiac death. It affects young subjects, predominantly males, with structurally normal hearts. The prevalence varies with ethnicity ranging from 1:2,000 to 1:100,000 in different parts of the world. Today, hundreds of variants in 17 genes have been associated with BrS of which mutations in *SCN5A*, coding for the cardiac voltage-gated sodium channel, accounts for the vast majority. Despite this, approximately 70% of BrS cases cannot be explained genetically with the current knowledge. Moreover, the monogenic role of some of the variants previously described as being associated with BrS has been questioned by their occurrence in about 4% (1:23) of the general population as found in NHLBI GO Exome Sequencing Project (ESP) currently including approximately 6500 individuals. If we add the variants described in the five newest identified genes associated with BrS, they appear at an even higher prevalence in the ESP (1:21). The current standard treatment of BrS is an implantable cardioverter-defibrillator (ICD). The risk stratification and indications for ICD treatment are based on the ECG and on the clinical and family history. In this review we discuss the genetic basis of BrS.

## Introduction

The Brugada syndrome (BrS) was first described as a clinical entity in 1992 (Brugada and Brugada, 1992). It is inherited in an autosomal dominant manner (Antzelevitch et al., 2005). BrS has traditionally been viewed as a consequence of comprised electrical function without structural abnormalities, although the latter has been reported (Coronel et al., 2005; Frustaci et al., 2005; Nademanee et al., 2011; Duthoit et al., 2012).

BrS is characterized by an ST-segment elevation in the right precordial ECG leads V_1_–V_3_. The most descriptive ECG changes have been described at consensus conferences, endorsed by Heart Rhythm Society (HRS) and European Heart Rhythm Association (EHRA) over the last decade (Antzelevitch et al., 2005). In a consensus report from 2012, two specific ECG patterns are found to be descriptive (Bayés de Luna et al., 2012).

The BrS ECG pattern is characterized by a coved type ST-segment elevation ≥2 mm followed by a negative T wave in at least one of the right precordial leads (V_1_–V_3_) in the presence or absence of a sodium channel-blocking agent (type 1 ECG). BrS is diagnosed when this is seen in conjunction with one of the following: ventricular tachycardia/fibrillation (VF/VT), a family history of sudden cardiac death (SCD) <45 years old, coved-type ECGs in family members, inducibility of VT with programmed electrical stimulation (PES), syncope or nocturnal agonal respiration (Antzelevitch et al., 2005).

Patients with BrS have an increased risk of SCD secondary to VT/VF (Antzelevitch et al., 2005). Different cohorts have reported different risk of developing VT/VF (Brugada et al., 2002; Priori et al., 2002; Eckardt et al., 2005). In the most updated and largest BrS population so far, the cardiac event rate per year was 0.5% in asymptomatic patients, 1.9% in patients with syncope and 7.7% in patients with aborted SCD. The median age of diagnosis was 45 ± 10 years (Probst et al., 2010). In general, men are affected 8–10 fold more often than women, probably due to gender differences in the expression of certain cardiac ion channels (Antzelevitch, 2006). Approximately 20% of Brugada patients also develop supraventricular arrhythmias with atrial fibrillation accounting for most of the cases (Antzelevitch et al., 2005).

The syndrome is estimated to be responsible for 4% of all sudden deaths (SD) and 20% of SD's among patients with structurally normal hearts. The prevalence is ranging between 1:2,000 and 1:100,000 (Hermida et al., 2000; Letsas et al., 2007; Gallagher et al., 2008; Sinner et al., 2009; Holst et al., 2012a) in different countries, and it is the most common cause of death, besides accidents, in men under 40 years in some parts of the world, e.g., in Thailand (Antzelevitch et al., 2005). The syndrome is probably underestimated due to the fact that the characteristic ECG-pattern often is dynamic and concealed and by the fact, that there are several differential diagnoses associated with elevated ST-segments in right precordial leads (Brugada et al., 2009). The characteristic ECG pattern can in some cases be unmasked by administration of sodium channel-blockers, by febrile state or by vasotonic agents. Indeed sodium channel blockers such as flecainide are used in the diagnosis of BrS (Antzelevitch et al., 2005).

Experimental studies have provided some understanding of the pathophysiological basis of the two main clinical characteristics; elevated coved ST-segment in V_1_–V_3_ and the increased risk of VT/VF (Yan and Antzelevitch, 1999). However, a consensus concerning the exact mechanism has not been established and there is an ongoing dispute as to whether BrS is a repolarization disorder, a depolarization disorder, or maybe both (Meregalli et al., 2005; Hoogendijk et al., 2010; Wilde et al., 2010). The central mechanism underlying the ECG pattern and arrhythmias, according to the repolarization hypothesis, is a more prominent transmural voltage-gradient in the early repolarization phase due to a more prominent I_to_ current in the epicardium compared to the endocardium of the right ventricle (Meregalli et al., 2005). Thus, in the epicardium, in the face of reduced sodium current, increased potassium current or reduced calcium current, complete loss of the phase 2 dome can occur. If this appears at some epicardial sites but not at others and a further heterogeneity between the epicardium and the endocardium occur, the result is an epicardial and transmural dispersion of repolarisation, respectively. This could lead to the development of a re-entry loop and premature beats already in the phase 2 of the on-going action potential (AP) that could further trigger VT/VF (Meregalli et al., 2005).

The reason why ST-segment elevation occurs in right precordial leads, and not left, has been suggested to be due to a more prominent I_to_ in the right ventricle than in the left (Di Diego et al., 1996). Accordingly, this current is believed to have a central role in BrS pathogenesis. This hypothesis has been tested by Calloe et al. (2009), who demonstrated that an I_to_ activator recapitulated the electrographic and arrhythmic manifestations of BrS.

The depolarization theory states that the substrate for the ECG changes and susceptibility of VT/VF is a slowing of conduction caused both by fibrosis in right ventricular outflow tract (RVOT) and a decreased I_Na_. This decrease in conduction velocity is more prominent in RVOT compared to the rest of the right ventricle which gives rise to the substrate for ECG changes and re-entry arrhythmias (Meregalli et al., 2005).

Recently Hoogendijk et al. stated that none of the proposed mechanism so far described has been irrefutably demonstrated in BrS patients. Therefore they suggested a unifying explanation, the so-called current-to-load mismatch. This hypothesis states that current-to-load mismatch caused by structural and functional abnormality could explain the ST-segment elevation and susceptibility to arrhythmias (Hoogendijk et al., 2010).

## Genetic basis of BrS

To date, 17 genes have been associated with BrS or BrS ECG phenotype (Table 1). *SCN5A* was the first gene to be associated with BrS and still represents the major gene in BrS pathogenesis. The individual genes associated with BrS are described in detail in the following.

**Table 1:** **Mutations in genes associated with Brugada syndrome**.

**BrS subtype**	**Gene name**	**Gene product**	**Ionic current**	**Functional effect**	**References**	**Incidence, %**
BrS1	*SCN5A*	Na_v_1.5	I_Na_	Loss-of-function	Chen et al., 1998	11–24 Kapplinger et al., 2010
BrS2	*GPD1-L*	G3PD1L	I_Na_	Loss-of-function	London et al., 2007	Rare Antzelevitch and Nof, 2008
BrS3	*CACNA1C*	Ca_v_1.2	I_Ca-L_	Loss-of-function	Antzelevitch et al., 2007	6–7 Antzelevitch and Nof, 2008
BrS4	*CACNB2*	Ca_v_β2	I_Ca-L_	Loss-of-function	Antzelevitch et al., 2007	4–5 Antzelevitch and Nof, 2008
BrS5	*SCN1B*	Na_v_β1	I_Na_	Loss-of-function	Watanabe et al., 2008	1–2 Antzelevitch and Nof, 2008
BrS6	*KCNE3*	MiRP2	I_to_/I_Ks_	Gain-of-function	Delpón et al., 2008	<1 Antzelevitch and Nof, 2008
BrS7	*SCN3B*	Na_v_β3	I_Na_	Loss-of-function	Hu et al., 2009	Probably rare
BrS8	*KCNH2*	hERG1	I_Kr_	Loss-of-function	Itoh et al., 2009; Verkerk et al., 2005	Probably rare
BrS9	*KCNJ8*	Kir6.1	I_KATP_	Gain-of-function	Medeiros-Domingo et al., 2010	Probably rare
BrS10	*CACNA2D1*	Ca_v_α_2_δ-1	I_Ca-L_	Not available	Burashnikov et al., 2010	Probably rare
BrS11	*RANGRF*	MOG1	I_Na_	Loss-of-function	Kattygnarath et al., 2011	Probably rare
BrS12	*KCNE5*	MiRP4	I_to_/I_Ks_	Gain-of-function	Ohno et al., 2011	Probably rare
BrS13	*KCND3*	K_v_4.3	I_to_	Gain-of-function	Giudicessi et al., 2011	Probably rare
BrS14	*HCN4*	HCN4	I_f_	Not available	Crotti et al., 2012	Probably rare
BrS15	*SLMAP*	SLMAP	I_Na_	Loss-of-function	Ishikawa et al., 2012	Probably rare
BrS16	*TRMP4*	TRMP4	NSC_Ca_	Both	Liu et al., 2013	6^*^
BrS17	*SCN2B*	Na_v_β2	I_Na_	Loss-of-function	Riuró et al., 2013	Probably rare

Subtypes listed chronologically.

NSC_Ca_, Calcium activated Non-Selective Cation channel.

* 6% of original cohort consisting of 248 BrS cases.

### BrS1 is associated with mutations in SCN5A

The *SCN5A* gene encodes the α-subunit of the voltage-dependent cardiac sodium channel, Na_v_1.5 (Gellens et al., 1992). Mutations in this gene in association with BrS were first described in 1998 by Chen et al. (1998). Since then more than 300 mutations in this gene have been associated with BrS.

Functional studies of many different mutations in the gene have been performed and they all lead to a reduction in net sodium current due to one or more of following reasons (Antzelevitch et al., 2005); (1) reduced current density due to failure of the sodium channel to express or defect trafficking of the channel (Baroudi et al., 2001; Valdivia et al., 2004; Pfahnl et al., 2007), (2) a shift in the voltage- and time-dependence of sodium channel current activation, inactivation or reactivation (Keller et al., 2005; Hsueh et al., 2009; Calloe et al., 2013), or (3) entry of the sodium channel into an intermediate state of inactivation from which it recovers relatively slower than normal (Bezzina et al., 1999; Veldkamp et al., 2000; Chiang et al., 2009). A number of knock-out mouse models support the central role of *SCN5A* in the pathogenesis of BrS (Killeen et al., 2008; Derangeon et al., 2012). Heterozygous knock-out mice have been shown to have compromised conduction velocity, impaired AV conduction and QRS prolongation. Furthermore during programmed ventricular electrical pacing two thirds of the mice developed ventricular tachyarrhythmias (Derangeon et al., 2012).

The arrhythmic potential of mutations in *SCN5A* is also emphasized by its involvement in other arrhythmic diseases such as LQTS, BrS, SIDS, cardiomyopathy and AF. Various *SCN5A* mutations are known to present with mixed phenotypes, a presentation known as cardiac sodium channel overlap syndrome (Darbar et al., 2008; Remme et al., 2008; Olesen et al., 2012a). This really emphasises the complexity of *SCN5A* gene mutations in BrS.

See supplementary Tables S1–S5 for a complete overview of all mutations in *SCN5A* associated with BrS.

### BrS2 is associated with mutations in GPD1L (see Table 2)

Weiss et al. (2002), linked a locus, close to but distinct from the *SCN5A* locus, to BrS in a large Italian family (Weiss et al., 2002). London et al. (2007) characterized the locus to be the glycerol-3-phosphat dehydrogenase 1-like, *GPD1-L* gene. There is 84% homology with the Glycerol-3-phosphate dehydrogenase protein, GPD, a dimer involved in the glycerol phosphate shuttle that transfers electrons from cytosolic NADH to the mitochondrial transport chain. The GPD1-L protein is highly expressed in the heart and is concentrated in the membrane fraction. London et al. found that an A280V mutation from a BrS patient in *GPD1-L* was linked to a 48% decrease in inward Na^+^ current and a marked decrease in surface expression of Na_v_1.5 (London et al., 2007).

**Table 2:** **Mutations in genes associated with BrS2-BrS17**.

**Subtype**	**Gene**	**Ionic current**	**Amino acid substitution**	**Functional effect**	**References**
BrS2	*GPD1L*	I_Na_	A280V	Loss-of-function	London et al., 2007
BrS3	*CACNA1C*	I_Ca-L_	A39V	Loss-of-function	Antzelevitch et al., 2007
			G490R	Loss-of-function	Antzelevitch et al., 2007
			E1115K	Loss-of-function	Burashnikov et al., 2010
			R1880Q	Loss-of-function	Burashnikov et al., 2010
			V2014I	Loss-of-function	Burashnikov et al., 2010
			D2130N	Loss-of-function	Burashnikov et al., 2010
BrS4	*CACNB2*	I_Ca-L_	S481L	Loss-of-function	Antzelevitch et al., 2007
			T11I	Loss-of-function	Cordeiro et al., 2009
			S143F	Loss-of-function	Burashnikov et al., 2010
			L399F	Loss-of-function	Burashnikov et al., 2010
			T450I	Loss-of-function	Burashnikov et al., 2010
			D538E	Loss-of-function	Burashnikov et al., 2010
			V340I	Not available	Crotti et al., 2012
			E499D	Not available	Crotti et al., 2012
BrS5	*SCN1B*	I_Na_	W179X	Loss-of-function	Watanabe et al., 2008
			R214Q	Loss-of-function	Holst et al., 2012b; Hu et al., 2012
			H162P	Not available	Holst et al., 2012b
			Q204R	Not available	Crotti et al., 2012
BrS6	*KCNE3*	I_to_/I_Ks_	R99H	Gain-of-function	Delpón et al., 2008
BrS7	*SCN3B*	I_to_/I_Ks_	L10P	Loss-of-function	Hu et al., 2009
			V110I	Loss-of-function	Ishikawa et al., 2013
BrS8	*KCNH2*	I_Kr_	G873S	Gain-of-function	Verkerk et al., 2005
			N985S	Gain-of-function	Verkerk et al., 2005
			R1135H	Gain-of-function	Itoh et al., 2009
BrS9	*KCNJ8*	I_KATP_	S422L	Gain-of-function	Medeiros-Domingo et al., 2010
BrS10	*CACNA2D1*	I_Ca-L_	D550Y	Not available	Burashnikov et al., 2010
			S709N	Not available	Burashnikov et al., 2010
			Q917H	Not available	Burashnikov et al., 2010
BrS11	*RANGRF*	I_Na_	E83D	Loss-of-function	Kattygnarath et al., 2011
BrS12	*KCNE5*	I_to_/I_Ks_	Y81H	Gain-of-function	Ohno et al., 2011
			D92E; E93X	Gain-of-function	Ohno et al., 2011
BrS13	*KCND3*	I_to_	L450F	Gain-of-function	Giudicessi et al., 2011
			G600R	Gain-of-function	Giudicessi et al., 2011
BrS14	HCN4	I_f_	S841L	Not available	Crotti et al., 2012
BrS15	*SLMAP*	I_Na_	V269I	Loss-of-function	Ishikawa et al., 2012
			E710A	Loss-of-function	Ishikawa et al., 2012
BrS16	*TRPM4*	NSC_Ca_	R144W	Not available	Liu et al., 2013
			A432T	Gain-of-function	Liu et al., 2013, 2010
			G555R	Not available	Liu et al., 2013
			G582S	Not available	Liu et al., 2013
			F773I	Not available	Liu et al., 2013
			P779R	Loss-of-function	Liu et al., 2013
			T873I	Gain-of-function	Liu et al., 2013
BrS17	*SCN2B*	I_Na_	D211G	Loss-of-function	Riuró et al., 2013

A mechanism by which *GPD1-L* mutations could affect Na_v_1.5 has been studied since by Liu et al. (2009). They, on the basis of the homology between *GPD* and *GPD1-L*, investigated whether the *GPD1-L*, as *GPD*, is involved in NAD-dependent energy metabolism and thereby, whether NAD(H) could regulate Na_v_1.5. Indeed they found that A280V-GPD1-L increased [NADH]_i_ and that this increase in [NADH]_i_ reduced *I*_*Na*_. This suggests a link between metabolism and *I*_*Na*_.

### BrS3 is associated with mutations in CACNA1C (see Table 2)

This gene encodes the α-subunit of the human L-type voltage-gated calcium channel, Ca_v_1.2 (Takimoto et al., 1997). Antzelevitch et al. (2007) identified an association between mutations in *CACNA1C* and BrS. In a Brugada cohort they found two missense mutations in two probands, G490R and A39V. The ECG of the affected patients revealed short QT interval. Both mutations occurred in highly conserved regions of the Ca_v_1.2 protein and both mutations led to a major loss-of-function in calcium channel activity. The loss-of-function caused by the A39V mutation was found to be caused by a trafficking defect (Antzelevitch et al., 2007).

### BrS4 is associated with mutations in CACNB2B (see Table 2)

This gene encodes the β-subunit of Ca_v_1.2, Ca_v_β2, which is involved in regulation of the gating process of *I*_*Ca−L*_, in increasing the *I*_*Ca−L*_ and in modulation of *I*_*Ca−L*_ traficking (Cornet et al., 2002; Catterall et al., 2005; Hedley et al., 2009).

Antzelevitch et al. (2007) identified a mutation in *CACNB2b* (S481L) in one proband with BrS as well as short QT. The S481L mutation was present in all 6 phenotype-positive and absent in all 4 phenotype-negative family members. The *I*_*Ca−L*_ was found to be reduced markedly. Hedley et al. (2009) suggested that the pathogenic mechanism for this mutation could be interference of the stimulatory role of Ca_v_β2 on *I*_*Ca−L*_, by the fact that the mutation is localized close to the Ca_v_1.2 binding domain.

In 2009, Cordeiro et al. associated a novel mutation in *CACNB2b* with BrS. They detected a missense mutation in *CACNB2b*, T11I. They found that the mutation led to an accelerated inactivation of the L-type Ca^2+^ channel. This change of kinetics resulted in a reduced depolarizing current contributing to the plateau phase of the epicardial AP (Cordeiro et al., 2009). Since, Burashnikov et al. (2010) have revealed additional mutations in *CACNB2b* (see Table 2).

### BrS5 is associated with mutations in SCN1B (see Table 2)

The gene encodes the β1-subunit of Na_v_1.5, Na_v_β1, which is translated into to isoforms; β1 and β1b. Functions attributed to the β-subunit include an increase in Na_v_1.5 expression at the cell surface, modulation of channel gating and voltage dependence, and a role in cell adhesion and recruitment of cytosolic proteins such as Ankyrin-G (Isom, 2001; Watanabe et al., 2008).

The association of mutations in *SCN1B* with BrS was first identified by Watanabe et al. (2008). They screened 282 probands with BrS and 44 patients with conduction disease. They identified one mutation (W179X (β1b)) in *SCN1B* in a patient with BrS ECG phenotype. The mutated form of *SCN1B* was not able to increase *I*_*Na*_ as normal. Recently, Holst *et al*. added further evidence for an association between mutations in *SCN1B* and BrS. Two mutations in two probands in *SCN1Bb* (H162P and R214Q) were identified from a cohort of 42 *SCN5A*-negative BrS patients. However, R214Q was also found in ESP; H162P was not (Holst et al., 2012b; Olesen et al., 2012b). Hu et al. (2012) investigated the functional consequence of the R214Q variant. When co-expressed with WT-*SCN5A* the mutant *SCN1Bb* induced a significant decrease in peak sodium current compared to WT-*SCN1Bb*. Interestingly, when co-expressed with WT-*KCND3*, the variant induced a greater *I*_*to*_, suggesting a combined loss of function of sodium channel current and gain of function of transient outward potassium current in BrS pathogenesis.

### BrS6 is associated with mutations in KCNE3 (see Table 2)

This gene encodes the protein MiRP2, one of five homologous β-subunits (*KCNE1-5*) of voltage gated potassium ion channels (Abbott et al., 2001; Hedley et al., 2009). The potassium channel complex, Kv:KCNE, is a heterohexameric structure consisting of four α-subunits and two *KCNE* peptides. The functional role of *KCNE* peptides, in general, is modulation of several potassium currents in the heart, for instance *I*_*to*_ and *I*_*Ks*_(Delpón et al., 2008). The association of mutations in *KCNE3* with BrS was identified by Delpón et al. (2008). They screened 105 probands with BrS and found a R99H missense mutation in one individual who were highly symptomatic with one aborted cardiac arrest and numerous appropriate shocks after ICD implantation. The family of this individual was examined and they found that 4/4 phenotype-positive and 0/3 phenotype-negative family members had the mutation. Co-transfection of R99H-*KCNE3* with *KCNQ1* produced no alteration in current magnitude or kinetics. Co-expressed with WT K_v_4.3, *I*_*to*_ channel, the mutation had a gain-of-function effect leading to an increase in peak current and an accelerated inactivation of *I*_*to*_. Overall, the mutation led to a significant increase in total charge carried by *I*_*to*_.

### BrS7 is associated with mutations in SCN3B (see Table 2)

This gene encodes the β3-subunit of the cardiac sodium channel, Na_v_β3 (Morgan et al., 2000). The functional attribution of Na_v_β3 is modulation of the channel gating of Na_v_1.5, similar to the β1-subunit, although with different kinetics (Morgan et al., 2000).

The association of *SCN3B* with BrS have been identified by Hu et al. (2009). They found a missense mutation (L10P) in an individual with BrS. The mutation led to a decrease in peak sodium current density, accelerated inactivation, and slowed reactivation compared to wild type. The L10P mutation has also been associated with lone atrial fibrillation (AF) suggesting an overlap in phenotypes (Olesen et al., 2011b). Recently, Ishikawa *et al*. reported another novel *SCN3B* mutation, V110I, in three of 178 unrelated Japanese BrS patients. (Ishikawa et al., 2013) The mutation was absent in 480 Japanese controls and displayed a loss-of-function effect due to impaired cell surface expression of Nav1.5.

### BrS8 is associated with mutations in KCNH2 (see Table 2)

The α-subunit of the rapid delayed rectifier channel (hERG1) is encoded by *KCNH2*. Verkerk et al. in 2005, identified two mutations (G873S and N985S) in two unrelated *SCN5A*-negative BrS patients. Functional investigation revealed an increase in the rectifying current, namely an increase in peak current during phase 0 and phase 1 of the ventricular AP. Through computer simulations this gain-of-function in *I*_*Kr*_ enhanced the susceptibility of loss of AP dome in right ventricular subepicardial myocytes, which is characteristic of BrS. G873S, however, was found in 2 of 500 unrelated Han Chinese controls suggesting that the variant has only a modifying role or is an innocent bystander. Further support for this interpretation is the fact that the glycine at position 873 is not conserved between human, mouse and rat (Verkerk et al., 2005). For this reason, they were not denoted as the first to associate mutations in *KCNH2* with BrS. In 2009, Itoh et al., as the “first”, identified a mutation (R1135H) in *KCNH2* in a 34-year old man with Brugada-type ECG and short QT interval. The mutation displayed a gain-of-function effect on *I*_*Kr*_(Itoh et al., 2009). Subsequently, Wilders and Verkerk, demonstrated, through computer simulations, that R1135H had the same consequence on AP as G873S and N985S (Wilders and Verkerk, 2010).

### BrS9 is associated with mutations in KCNJ8 (see Table 2)

This gene encodes the cardiac K_ATP_ channel, Kir6.1. The Kir6.1 channel facilitates a non-voltage-gated inwardly rectifying potassium current, leading to a shortening of the AP duration under conditions of metabolic stress (Delaney et al., 2012).

Medeiros-Domingo et al. (2010) found a mutation (S422L) in *KCNJ8* in a patient with a flecainide induced type 1 ECG pattern. Electrophysiological the mutation displayed a gain-of-function consequence on K_ATP_. Subsequently, Barajas-Martínez et al. (2012) identified the same mutation in three other BrS patients. When *KCNJ8*-S422L was co-expressed with the wild type regulatory SUR2A, it showed a twofold gain-of-function on *I*_*K, ATP*_. Furthermore, the mutant channel displayed a reduced sensitivity to ATP, pointing to incomplete closing of the channel under normoxic conditions.

### BrS10 is associated with mutations in CACNA2D1 (see Table 2)

*CACNA2D1* encodes the α_2_δ-subunit of the voltage-dependent calcium channel and has been found to share similar functional properties with Ca_v_β2 (Gurnett et al., 1996; Hobom et al., 2000). Burashnikov *et al*. identified three different missense mutations in *CACNA2D1* (S709N, D550Y and Q917H) in three BrS patients from a cohort consisting of 205 patients with BrS, short QT, idiopathic ventricular fibrillation (IVF) and early repolarisation syndrome. However, in two of the three patients, additional mutations in genes recognized as being associated with BrS were identified. Unfortunately, the authors did not investigate the electrophysiological consequence of the three missense mutations. New mutations in *CACNB2b* and *CACNA1C* were also detected in this cohort (see Table 2) (Burashnikov et al., 2010).

### BrS11 is associated with mutations in RANGRF (see Table 2)

Kattygnarath et al. (2011) reported the gene *RANGRF*, encoding MOG1 (a protein important for the trafficking of *SCN5A* to the cell membrane), as a new BrS gene. They identified a missense mutation E83D in a BrS patient that dominant-negatively compromised the sodium current. The mutation was not found in 281 control subjects. Olesen et al. identified another MOG1 variant, E61X, in both AF patients and healthy controls indicating that a person may have complete loss of one MOG1 allele without having any signs of disease (Olesen et al., 2011a). Genetic variants that compromise the MOG1 protein are therefore more likely to increase the susceptibility of BrS, rather than to be the major genetic susceptibility variant.

### BrS12 is associated with mutations in KCNE5 (see Table 2)

This gene encodes one of the regulatory β-subunits of the *I*_*to*_/*I*_*Ks*_ channels mentioned under BrS6. In 2011, Ohno et al. identified two novel variants (Y81H and D92E;E93X) in *KCNE5* in a Japanese cohort consisting of 205 patients with BrS or IVF (Ohno et al., 2011). Three probands comprised the Y81H variant, one the D92E;E93X variant. In 300 unrelated healthy Japanese controls, Y81H was identified in 3 women, and D92E; E93X was absent. All four probands were symptomatic and received an ICD. When co-expressed with *KCND3* (α-subunit of *I*_*to*_), the mutant channels significantly increased *I*_*to*_ compared to wild type, displaying a gain-of-function effect. Stimulation study revealed that the two variants induced altered ventricular AP profiles. This could provide the likelihood of a proarrhythmic substrate. Another interesting notion drawn by the authors is that *KCNE5* is located on chromosome X. This could in part explain the gender difference seen in prevalence. Indeed, the male phenotype in the study by Ohno et al. was more severe.

### BrS13 is associated with mutations in KCND3 (see Table 2)

*KCND3* encodes the α-subunit of *I*_*to*_, K_v_4.3, a voltage-gated potassium channel expressed in heart. In 2011, two novel mutations (L450F and G600R) were identified in two unrelated BrS patients (Giudicessi et al., 2011). Both mutations were absent in 1560 reference alleles. Co-expression of K_v_4.3 mutants with KChIP2-WT revealed a significant increase in I_to_ current density compared with WT-K_v_4.3. Moreover, the two mutations induced loss of AP dome in RV epicardial myocytes, demonstrated by computer simulations, providing the arrhythmic substrate for BrS phenotype (Giudicessi et al., 2011).

### BrS14 is associated with mutations in HCN4 (see Table 2)

*HCN4* encodes the hyperpolarisation-activated cyclic nucleotide-gated channel 4 which is a pacemaker channel responsible for the funny current (*I*_*f*_). Mutations in this gene has formerly been associated with sinus node dysfunction (Ueda et al., 2004). Recently, Crotti *et al*. identified a mutation (S841L) in *HCN4* in one proband of 129 unrelated BrS patients (Crotti et al., 2012). The mutation was absent in ≥1400 ethnicity matched reference alleles and in publicly available databases. The functional effect of this mutation has not been assessed and therefore, before drawing any conclusion in BrS pathogenesis, this gene has to be investigated more thoroughly.

### BrS15 is associated with mutations in SLMAP (see Table 2)

*SLMAP* encodes the sarcolemmal membrane-associated protein, a component of T-tubules and sarcoplasmic reticulum which is involved in excitation-contraction coupling in cardiomyocytes (Ishikawa et al., 2012). Ventricular arrhythmias have previously been linked to mutations in proteins involved in excitation-contraction coupling (Priori et al., 2001). Ishikawa et al. (2012) recently reported two mutations (V269I and E710A) in 190 unrelated BrS patients. In cell lines the two mutations were shown to reduce cell surface expression of Na_v_1.5 resulting in decreased peak sodium current density. In line with this, the investigators demonstrated that silencing the two *SLMAP* mutants rescued the decreased surface expression of Na_v_1.5.

### BrS16 is associated with mutations in TRPM4 (see Table 2)

The *TRPM4* gene encodes the transient receptor potential melastatin protein number 4 which is a calcium-activated non-selective cation channel (NSC_Ca_) that mediates transport of monovalent cations across membranes, thereby depolarizing the membrane. Mutations in this gene have been associated with cardiac conduction blocks (Kruse et al., 2009; Liu et al., 2010; Stallmeyer et al., 2012) and recently, Liu et al. (2013) associated mutations in *TRPM4* with BrS. In 248 BrS cases, the investigators identified 7 mutations absent in approximately 14,000 control alleles. Functional characterization of selected mutations revealed both a decrease in *TRPM4* expression (P779R) and an increase in expression (T873I) suggesting that both loss- and gain-of-function mutations in this gene may lead to BrS.

### BrS17 is associated with mutations in SCN2B (see Table 2)

The *SCN2B* gene encodes the β2-subunit of the cardiac sodium channel. The association of *SCN2B* with BrS have just recently been identified by Riuró et al. (2013). They found a missense mutation (A211G) in an individual with BrS. The mutation was absent in 500 control alleles and available databases. The mutation led to a significant reduction in sodium current density when co-expressed with Na_v_1.5 compared to wild type. This reduction was shown to be due to a reduced Na_v_1.5 cell surface expression (Riuró et al., 2013).

## Bioinformatic re-evaluation of variants

With the recently published exome data from the NHLBI GO Exome Sequencing Project (ESP), knowledge regarding genetic variation in the general population have become available [Exome Variant Server, NHLBI GO (ESP)]. In ESP, next-generation sequencing has been carried out for all protein-coding regions in approximately 6500 persons from different population studies. Risgaard et al. (2013) have, by using these data, found a high genotype prevalence of 1:23 in the ESP of genetic variants in twelve genes (*SCN5A, GPD1L, CACNA1C, CACNB2, SCN1B, KCNE3, SCN3B, KCNH2, CACNA2D1, MOG1, KCND3*, and *KCNJ8*) previously associated with BrS. This is a very high prevalence compared to the prevalence of BrS in the general population ranging between 1:2,000 and 1:100,000 (Hermida et al., 2000; Letsas et al., 2007; Gallagher et al., 2008; Sinner et al., 2009; Holst et al., 2012a). Moreover, in a synergistic use of prediction analysis using ≥3 prediction tools, 47% of the variants found in ESP were predicted pathogenic compared to 75% of the variants not found in ESP (*p* < 0.0001). These data definitely questions the pathogenic role of some of the previously BrS-associated variants. A limitation in the study is that there are no clinical data on the persons in ESP. However, Refsgaard et al. have recently conducted a number of studies that indicate, that the exome database is indeed representative for genetic variation in healthy subject (Refsgaard et al., 2012; Andreasen et al., 2013a,b). Moreover, none of the studies in ESP specifically included patients with channelopathies and at least two studies excluded such patients (Refsgaard et al., 2012).

We investigated the prevalence in ESP of the genes not investigated by Risgaard et al. (2013), BrS subtypes 12 and 14-17. The *KCNE5, SCN2B* and *SLMAP* mutations were not found in ESP. The *HCN4* mutation (S841L) was found in 3 out of 4289 European American (EA) individuals. The *TRPM4* mutation R144W was present in 1 of 2199 Afro-American (AA) individuals, A432T in 9 of 4291 AA, and G582S in 9 of 4291 EA. The rest of the *TRPM4* mutations were not present in ESP [Exome Variant Server, NHLBI GO (ESP)]. If we add the variants found in the five newest identified genes associated with BrS, this corresponds to an even higher genotype prevalence of 1:21 (296:6258) in ESP.

## Treatment

### Implantable cardioverter defibrillator—ICD

ICD is the only widely accepted treatment of BrS thus far (Brugada et al., 1999, 2000). In 2003, a second consensus conference was held which focused on risk stratifications and approaches to therapy (Antzelevitch et al., 2005). This consensus report stated the recommendations for ICD implantation.

Sarkozy et al. (2007) studied the effectiveness of ICD treatment in a retrospective study. 47 high risk Brugada patients (mean age: 44 ± 15 years) with ICD were included. During a mean follow-up of 47.5 months, seven patients had appropriate shocks for potentially life-threatening ventricular arrhythmias. However, seventeen patients received inappropriate shocks due to shocks for sinus tachycardia and atrial arrhythmias, which is common in Brugada patients.

A multicenter study by Sacher et al. (2006) showed the same pattern. In 220 BrS patients with ICD (mean follow up >3 years) 8% experienced appropriate shocks and 20% inappropriate shocks. Overall, complications occurred in 28% of the patients.

In a just published article, Miyazaki et al. (2013) also investigated the prevalence of ICD-related complications. In 41 BrS patients and during a median follow-up of 76 months, 15 patients (37%) experienced adverse effects after ICD implantation. This includes complications in 8 (20%) and inappropriate shocks in 10 (24%). Appropriate shocks were detected in 5 patients (12%), (please keep in mind that some patients experience more than one adverse effect). In a nationwide study by Holst et al. (2012a), 26% experienced appropriate shocks and 8% experienced inappropriate shocks during a median follow-up of 47 months in 35 definite BrS patients. The difference in rate of appropriate and inappropriate shocks compared to the three other studies could be due to a more severe phenotype of patients included and due to difference in ICD discrimination algorithms as suggested by Holst et al. (2012a).

A pharmacological approach with fewer complications is obviously desirable and there is a growing effort to define such a safe and efficient treatment for this specific syndrome.

### Pharmacological therapy

Loss-of-function mutations are responsible for the vast majority of BrS incidents. This makes it more difficult in regard to a pharmacological therapy as it is difficult to compensate for the missing allele. However some substances may be beneficial. The objective is to rebalance the inward and outward currents during the AP and thereby restoring electrical homogeneity (Brugada et al., 2009).

The prominent *I*_*to*_ in the right ventricle is thought to have a central role in the pathogenesis of BrS, so a drug like quinidine that inhibits *I*_*to*_ (Imaizumi and Giles, 1987) has been suggested to have a therapeutic value in BrS (Yan and Antzelevitch, 1999).

Hermida et al. (2004) observed that hydroquinidine therapy prevented VT/VF inducibility in 22 out of 29 asymptomatic patients with BrS and inducible arrhythmia, as well as VT/VF recurrence in four BrS patients with multiple ICD shocks. Belhassen et al. (2004) reported that quinidine bisulfate prevented VF induction in 22 of 25 BrS patients. All 25 patients had inducible VF before treatment. However, administration of quinidine was associated with a 36% incidence of side-effects that resolved after drug discontinuation. In general, disadvantages of oral quinidine include gastrointestinal side-effects, as observed by Belhassen et al. (2004), and proarrhythmic side-effects (QT prolongation), as observed by Hermida et al. (2004). The latter is probably due to a quinidine block of *I*_*Kr*_ and *I*_*Ks*_, however this side effect is rare (Antzelevitch and Nof, 2008). In a recent study by Márquez et al. (2012) the authors investigate the long-term efficacy of low doses quinidine on malignant arrhythmias in BrS patients. A total of twenty patients, of whom seventeen patients had an ICD, were included. In all but three cases, quinidine effectively suppressed arrhythmic events corresponding to an efficacy of quinidine on 85%. All patients tolerated the medication well.

Taken together the data suggest that preventive treatment by quinidine may be an alternative or complimentary strategy to ICD in BrS patients, both in the short and long term. A more *I*_*to*_ selective compound that does not permeate the blood-brain-barrier would in theory be the optimal treatment. For other possible beneficial agents please see Márquez et al. (2007) and Minoura et al. (2013).

## Discussion and perspectives

Presently over 300 mutations in 17 genes have been associated with BrS or BrS ECG phenotype, in contrast to 5 years ago where only mutations in the *SCNA5* gene were associated with BrS. The knowledge about BrS associated mutations is therefore rapidly increasing and this could potentially make genetic screening important in future. The intention would be to use this knowledge in risk stratification, as some asymptomatic BrS patients have an appreciable risk of arrhythmia (Probst et al., 2010). The rapidly declining cost of multi-gene screening by Next Generation Sequencing adds to the rationale. A study by Meregalli et al. (2009) reveal an association between the type of *SCN5A* mutation and the clinical severity. They compared groups having either missense mutations or mutations leading to premature truncation of the protein. They found that the disease phenotype was more severe in the patients with large *I*_*Na*_ reduction than in those with small *I*_*Na*_ reduction (truncation versus missense), as evidenced by larger proportions of patients with syncope and SCD. Sommariva et al. (2013) recently demonstrated that *SCN5A* mutation carriers had a significantly increased risk of major arrhythmic event compared with non-carriers in a BrS cohort. In addition they established an association of five polymorphisms with major arrhythmic event. Crotti et al. (2012) recently conducted a comprehensive mutational analysis of twelve BrS genes in a large BrS cohort. They did not detect any significant difference in mutation yield between those patients with definite BrS and those patients only displaying a type 1 ECG pattern. On this basis, the authors argue that genetic testing should additionally be conducted in patients displaying only type 1 ECG pattern. Secondly, they demonstrated that inclusion of the minor BrS susceptibility genes (genes other than *SCN5A*) in genetic testing, only minimally affected the sensitivity of the test. Therefore, these genes should only be screened under special circumstances.

These data may show some promise for the use of genetic data in risk stratification regarding clinical outcome in BrS patients. However, the scientific community is increasingly focusing on separating genetic noise from true pathogenic mutations. Risgaard et al. (2013) reported a high prevalence (1:23) of previously BrS-associated variants in ESP. If we added the variants described in the five newest identified genes associated with BrS, they appeared at an even higher prevalence in the ESP (1:21). These data definitely questions the pathogenic role of some of the previously BrS-associated variants. With regard to deletions and insertions in *SCN5A* (Tables S1–S5) these are more likely susceptibility mutations, as also evidenced by their lack of presence in ESP.

In a HRS/EHRA consensus document, screening is recommended in family members and relatives following the identification of a BrS-causative mutation in an index case (Ackerman et al., 2011). If the clinician should perform risk stratifications based on genetic screening it is important that variants being associated with BrS are truly pathogenic. An index patient with definite BrS but a false-positive variant might have another true pathogenic variant that is not found. This could lead to misdiagnosis of family members with clinical consequences.

Another important motive for identifying the important susceptibility mutations by genetically screening BrS patients could be the tailoring of specific drugs to specific mutations as personal medicine. Presently, the rationale behind the pharmacological approach is to rebalance the inward and outward currents during the AP, regardless of the underlying mutation. Maybe in the future, it could be possible to treat the different BrS types (1–17) differently, according to their mutational consequences, although the disease entity is so small that the drugs will most likely not be developed for this indication in the first case. For instance, in the study done by Liu et al. (2009), they found that the NADH-induced decrease in *I*_*Na*_ could be antagonized by externally or internally applied NAD^+^. This result suggests that drugs that increase the availability of NAD^+^ may be a future treatment strategy for BrS2. Teng et al. (2009) found that a *SCN5A* non-sense mutation (W822X) associated with BrS effectively could be suppressed by read-through enhancing agents, thereby restoring the expression of normal length sodium channels. This holds promising for all non-sense mutations associated with BrS. Chakrabarti et al. (2013) recently showed that overexpression of *MOG1* effectively rescued the trafficking defect and the impaired plasma membrane expression of Na_v_1.5 caused by the mutations D1275N and G1743, respectively. These data suggests that MOG1-enabled trafficking of Nav1.5 to plasma membrane may serve as a novel therapy for BrS patients with loss-of-function mutations in Nav1.5.

Even though over 300 mutations in 17 genes have been associated with BrS, approximately 70% of BrS incidents cannot be explained genetically at present. The causes may have to be found in epigenetic regulation or in other mechanisms than ion channel mutations. For instance methylation of promoters or mutations in microRNA binding sites as is has been shown for LQTS1 (Amin et al., 2012).

There is a need for an alternative strategy to ICD therapy. Firstly, because ICD treatment is prohibitively expensive in many parts of the world (Brugada et al., 2009). This is the case in Thailand where the prevalence is exceptionally high. Secondly, the high incidence of side-effects/complications associated with ICD (Sacher et al., 2006; Sarkozy et al., 2007; Miyazaki et al., 2013). And thirdly, BrS has been linked to sudden death infant syndrome (Van Norstrand et al., 2007) and ICD therapy in children is challenging in general. The high risk of complications reported in adults is likely to be worse in children (Antzelevitch and Nof, 2008).

The incentive for developing a good pharmacological paradigm is evident. Quinidine is yet the best alternative to ICD and has proven effective in small case series. However, a clear need exists for a large randomized clinical controlled trial to assess the effectiveness of quinidine in BrS patients.

### Conflict of interest statement

The authors declare that the research was conducted in the absence of any commercial or financial relationships that could be construed as a potential conflict of interest.
