# Supplementary material for: The genetic component of Brugada syndrome
Source: Front Physiol. 2013 Jul 15;4:179. doi: 10.3389/fphys.2013.00179 (PMC3710955; doi:10.3389/fphys.2013.00179)
Supplement: Supplementary file 1 [file DataSheet1.DOCX]

**Supplementary table S1. *SCN5A* missense/nonsense mutations associated with BrS.**

| **Amino acid substitution** | **Grantham** | **Polyphen** | **SIFT** | **CAS** | **Agreement of ≥3 *in silico* tools in damaging prediction** | **Reference** |
| --- | --- | --- | --- | --- | --- | --- |
| p.M1I | 10 | POS | DAM | Yes | Yes | Kapplinger (2010) Heart Rhythm **7,** 33 |
| p.A2T | 58 | POS | DAM | Yes | Yes | García-Castro (2010) Rev Esp Cardiol **63,** 856 |
| p.R18Q | 43 | PRO | TOL | No | No | Kapplinger (2010) Heart Rhythm **7,** 33 |
| p.R27H | 29 | POS | DAM | No | No | Priori (2002) Circulation **105,** 1342 |
| p.G35S | 56 | BEN | TOL | No | No | Levy-Nissenbaum (2001) Genet Test **5,** 331 |
| p.R43X | NA | NA | NA | NA | NA | Crotti (2009) Hum Genet **126** 339 |
| p.Q55X | NA | NA | NA | NA | NA | Makita (2007) Heart Rhythm **4,** 516 |
| p.N70K | 94 | PRO | DAM | No | No | Kapplinger (2010) Heart Rhythm **7,** 33 |
| p.D84N | 23 | PRO | DAM | Yes | Yes | Kapplinger (2010) Heart Rhythm **7,** 33 |
| p.F93S | 155 | PRO | DAM | Yes | Yes | Kapplinger (2010) Heart Rhythm **7,** 33 |
| p.I94S | 142 | PRO | DAM | No | Yes | Kapplinger (2010) Heart Rhythm **7,** 33 |
| p.V95I | 29 | PRO | DAM | Yes | Yes | Liang (2006) Zhonghua Xin Xue Guan Bing Za Zhi **34,** 616 |
| p.R104Q | 43 | PRO | DAM | Yes | Yes | Levy-Nissenbaum (2001) Genet Test **5,** 331 |
| p.R104W | 101 | PRO | DAM | Yes | Yes | Kapplinger (2010) Heart Rhythm **7,** 33 |
| p.N109K | 94 | BEN | TOL | No | No | Amin (2009) Circ Arrhythm Electrophysiol **2,** 531 |
| p.R121Q | 43 | PRO | DAM | Yes | Yes | Kapplinger (2010) Heart Rhythm **7,** 33 |
| p.R121W | 101 | PRO | DAM | Yes | Yes | Hedley (2009) Hum Mutat **30,** 1256 |
| p.A124D | 126 | PRO | DAM | Yes | Yes | Moreau (2012) Front Pharmacol **3,** 62 |
| p.K126E | 56 | POS | DAM | Yes | Yes | Vatta (2002) Mol Genet Metab **75,** 317 |
| p.L136P | 98 | POS | DAM | No | No | Yokokawa (2007) Am J Cardiol **100,** 649 |
| p.V146M | 21 | PRO | TOL | Yes | No | Kapplinger (2010) Heart Rhythm **7,** 33 |
| p.Y159X | NA | NA | NA | NA | NA | Kapplinger (2010) Heart Rhythm **7,** 33 |
| p.E161Q | 29 | PRO | DAM | Yes | Yes | Kapplinger (2010) Heart Rhythm **7,** 33 |
| p.E161K | 56 | PRO | DAM | Yes | Yes | Smits (2002) J Am Coll Cardiol **40,** 350 |
| p.K175N | 94 | PRO | DAM | Yes | Yes | Kapplinger (2010) Heart Rhythm **7,** 33 |
| p.A178G | 60 | PRO | DAM | Yes | Yes | Kapplinger (2010) Heart Rhythm **7,** 33 |
| p.R179X | NA | NA | NA | NA | NA | Kawamura (2009) Circ J **73,** 584 |
| p.C182R | 180 | PRO | DAM | Yes | Yes | Kapplinger (2010) Heart Rhythm **7,** 33 |
| p.A185V | 64 | POS | TOL | No | No | Kapplinger (2010) Heart Rhythm **7,** 33 |
| p.T187I | 89 | PRO | DAM | Yes | Yes | Makiyama (2005) J Am Coll Cardiol **46,** 2100 |
| p.W193X | NA | NA | NA | NA | NA | Kapplinger (2010) Heart Rhythm **7,** 33 |
| p.A204V | 64 | PRO | DAM | Yes | Yes | Kapplinger (2010) Heart Rhythm **7,** 33 |
| p.L212Q | 113 | PRO | DAM | Yes | Yes | Kapplinger (2010) Heart Rhythm **7,** 33 |
| p.V223L | 32 | PRO | DAM | No | No | Kapplinger (2010) Heart Rhythm **7,** 33 |
| p.R225W | 101 | PRO | DAM | Yes | Yes | Hedley (2009) Hum Mutat **30,** 1256 |
| p.A226V | 64 | PRO | DAM | Yes | Yes | Priori (2002) Circulation **105,** 1342 |
| p.I230V | 29 | PRO | TOL | Yes | No | Priori (2002) Circulation **105,** 1342 |
| p.K249X | NA | NA | NA | NA | NA | Kapplinger (2010) Heart Rhythm **7,** 33 |
| p.Q270K | 53 | PRO | DAM | Yes | Yes | Kapplinger (2010) Heart Rhythm **7,** 33 |
| p.L276Q | 113 | PRO | DAM | Yes | Yes | Yokokawa (2007) Am J Cardiol **100,** 649 |
| p.H278D | 81 | POS | DAM | No | No | Kapplinger (2010) Heart Rhythm **7,** 33 |
| p.R282C | 180 | PRO | DAM | Yes | Yes | Kapplinger (2010) Heart Rhythm **7,** 33 |
| p.R282H | 29 | PRO | DAM | Yes | Yes | Priori (2002) Circulation **105,** 1342 |
| p.G292S | 56 | BEN | TOL | No | No | Niimura (2004) Circ J **68,** 740 |
| p.V294M | 21 | PRO | TOL | Yes | No | Priori (2002) Circulation **105,** 1342 |
| p.V300I | 29 | BEN | TOL | No | No | Kapplinger (2010) Heart Rhythm **7,** 33 |
| p.W301X | NA | NA | NA | NA | NA | Kotta (2010) Int J Cardiol **145,** 45 |
| p.L315P | 98 | POS | TOL | No | No | Kapplinger (2010) Heart Rhythm **7,** 33 |
| p.K317N | 94 | BEN | TOL | Yes | No | Yi (2003) Di Yi Jun Yi Da Xue Xue Bao **23,** 1139 |
| p.G319S | 56 | BEN | TOL | Yes | No | Priori (2002) Circulation **105,** 1342 |
| p.T320N | 65 | PRO | TOL | Yes | No | Kapplinger (2010) Heart Rhythm **7,** 33 |
| p.L325R | 102 | BEN | DAM | Yes | Yes | Keller (2005) Cardiovasc Res **67,** 510 |
| p.P336L | 98 | PRO | DAM | Yes | Yes | Cordeiro (2006) Circulation **114,** 2026 |
| p.G351D | 94 | PRO | DAM | Yes | Yes | Kapplinger (2010) Heart Rhythm **7,** 33 |
| p.G351V | 109 | PRO | DAM | Yes | Yes | Vatta (2002) Mol Genet Metab **75,** 317 |
| p.T353I | 89 | PRO | DAM | Yes | Yes | Pfahnl (2007) Heart Rhythm **4,** 46 |
| p.D356N | 23 | PRO | DAM | Yes | Yes | Makiyama (2005) J Am Coll Cardiol **46,** 2100 |
| p.R367C | 180 | PRO | DAM | Yes | Yes | Smits (2002) J Am Coll Cardiol **40,** 350 |
| p.R367L | 102 | PRO | DAM | Yes | Yes | Kapplinger (2010) Heart Rhythm **7,** 33 |
| p.M369K | 95 | PRO | DAM | Yes | Yes | Smits (2002) J Am Coll Cardiol **40,** 350 |
| p.W374G | 184 | PRO | DAM | Yes | Yes | Kapplinger (2010) Heart Rhythm **7,** 33 |
| p.R376H | 29 | PRO | DAM | Yes | Yes | Rossenbacker (2004) Heart Rhythm **1,** 610 |
| p.G386R | 125 | PRO | DAM | Yes | Yes | Kapplinger (2010) Heart Rhythm **7,** 33 |
| p.G386E | 98 | PRO | DAM | Yes | Yes | Kapplinger (2010) Heart Rhythm **7,** 33 |
| p.V396A | 64 | PRO | DAM | Yes | Yes | Kapplinger (2010) Heart Rhythm **7,** 33 |
| p.V396L | 32 | PRO | DAM | Yes | Yes | Kapplinger (2010) Heart Rhythm **7,** 33 |
| p.N406S | 46 | PRO | DAM | Yes | Yes | Itoh (2005) J Cardiovasc Electrophysiol **16,** 486 |
| p.Q419X | NA | NA | NA | NA | NA | Kapplinger (2010) Heart Rhythm **7,** 33 |
| p.E439K | 56 | PRO | TOL | Yes | No | Kapplinger (2010) Heart Rhythm **7,** 33 |
| p.E473X | NA | NA | NA | NA | NA | Baroudi (2004) Can J Cardiol **20,** 425 |
| p.D501G | 94 | PRO | TOL | Yes | No | Kapplinger (2010) Heart Rhythm **7,** 33 |
| p.R526H | 29 | BEN | TOL | No | No | Kapplinger (2010) Heart Rhythm **7,** 33 |
| p.R535X | NA | NA | NA | NA | NA | Smits (2002) J Am Coll Cardiol **40,** 350 |
| p.F543L | 22 | PRO | TOL | Yes | No | Kapplinger (2010) Heart Rhythm **7,** 33 |
| p.A551T | 58 | POS | TOL | No | No | Lai (2005) J Hum Genet **50,** 490 |
| p.G552R | 125 | PRO | TOL | Yes | Yes | Kapplinger (2010) Heart Rhythm **7,** 33 |
| p.E555K | 56 | PRO | TOL | Yes | No | Takahata (2003) Life Sci **72,** 2391 |
| p.L567Q | 113 | PRO | TOL | No | No | Priori (2000) Lancet **355,** 808 |
| p.Q573X | NA | NA | NA | NA | NA | Kapplinger (2010) Heart Rhythm **7,** 33 |
| p.A586T | 58 | BEN | TOL | No | No | Nakajima (2011) Int Heart J **52,** 27 |
| p.R620C | 180 | POS | TOL | No | No | Kapplinger (2010) Heart Rhythm **7,** 33 |
| p.R620H | 29 | BEN | TOL | No | No | Calloe (2013) Circ Arrhythm Electrophysiol **6,** 1 |
| p.T632M | 81 | PRO | TOL | Yes | No | Kapplinger (2010) Heart Rhythm **7,** 33 |
| p.P640A | 27 | BEN | TOL | Yes | No | Kapplinger (2010) Heart Rhythm **7,** 33 |
| p.A647D | 126 | BEN | TOL | Yes | No | Kapplinger (2010) Heart Rhythm **7,** 33 |
| p.R661W | 101 | PRO | DAM | Yes | Yes | Kapplinger (2010) Heart Rhythm **7,** 33 |
| p.H681P | 77 | BEN | DAM | No | No | Priori (2002) Circulation **105,** 1342 |
| p.C683G | 159 | BEN | DAM | Yes | Yes | Kapplinger (2010) Heart Rhythm **7,** 33 |
| p.E698X | NA | NA | NA | NA | NA | Kapplinger (2010) Heart Rhythm **7,** 33 |
| p.P717L | 98 | BEN | DAM | Yes | No | Kapplinger (2010) Heart Rhythm **7,** 33 |
| p.V728I | 29 | BEN | TOL | Yes | No | Garcia-Molina (2012) Clin Genet. 17 |
| p.A735E | 107 | BEN | DAM | Yes | Yes | Priori (2002) Circulation **105,** 1342 |
| p.A735T | 58 | BEN | DAM | Yes | No | García-Castro (2010) Rev Esp Cardiol **63,** 856 |
| p.E746K | 56 | BEN | TOL | No | No | Kapplinger (2010) Heart Rhythm **7,** 33 |
| p.G752R | 125 | PRO | DAM | Yes | Yes | Potet (2003) J Cardiovasc Electrophysiol **14,** 200 |
| p.G758E | 98 | POS | DAM | Yes | Yes | Kapplinger (2010) Heart Rhythm **7,** 33 |
| p.M764R | 91 | POS | DAM | Yes | Yes | Kapplinger (2010) Heart Rhythm **7,** 33 |
| p.P773S | 74 | POS | DAM | Yes | Yes | Kapplinger (2010) Heart Rhythm **7,** 33 |
| p.V789I | 29 | PRO | DAM | Yes | Yes | Kapplinger (2010) Heart Rhythm **7,** 33 |
| p.R808C | 180 | PRO | DAM | Yes | Yes | Kotta (2010) Int J Cardiol **145,** 45 |
| p.R811H | 29 | POS | DAM | Yes | Yes | Calloe (2013) Circ Arrhythm Electrophysiol **6,** 1 |
| p.R814Q | 43 | PRO | DAM | Yes | Yes | Frigo (2007) Europace **9,** 391 |
| p.W822C | 215 | PRO | DAM | Yes | Yes | Crotti (2012) J Am Coll Cardiol **epub,** epub |
| p.W822X | NA | NA | NA | NA | NA | Keller (2005) Can J Cardiol **21,** 925 |
| p.S835L | 145 | PRO | DAM | Yes | Yes | Niimura (2004) Circ J **68,** 740 |
| p.L839P | 98 | PRO | DAM | Yes | Yes | Probst (2006) J Cardiovasc Electrophysiol **17,** 97 |
| p.F851L | 22 | PRO | DAM | Yes | Yes | Priori (2002) Circulation **105,** 1342 |
| p.E867Q | 29 | PRO | DAM | Yes | Yes | Kapplinger (2010) Heart Rhythm **7,** 33 |
| p.E867X | NA | NA | NA | NA | NA | Smits (2002) J Am Coll Cardiol **40,** 350 |
| p.R878C | 180 | PRO | DAM | Yes | Yes | Crotti (2008) Hum Genet **123** 542 |
| p.R878H | 29 | PRO | DAM | Yes | Yes | Kapplinger (2010) Heart Rhythm **7,** 33 |
| p.H886P | 77 | POS | DAM | Yes | Yes | Kapplinger (2010) Heart Rhythm **7,** 33 |
| p.I890T | 89 | POS | DAM | Yes | Yes | Tarradas (2013) PLoS One **1,** 8 |
| p.F892I | 21 | POS | DAM | Yes | Yes | Priori (2002) Circulation **105,** 1342 |
| p.R893C | 180 | PRO | DAM | Yes | Yes | Kapplinger (2010) Heart Rhythm **7,** 33 |
| p.R893H | 29 | PRO | DAM | Yes | Yes | Kapplinger (2010) Heart Rhythm **7,** 33 |
| p.C896S | 112 | BEN | DAM | Yes | Yes | Priori (2002) Circulation **105,** 1342 |
| p.E901K | 56 | PRO | DAM | Yes | Yes | Kapplinger (2010) Heart Rhythm **7,** 33 |
| p.S910L | 145 | PRO | DAM | No | Yes | Priori (2002) Circulation **105,** 1342 |
| p.C915R | 180 | PRO | DAM | Yes | Yes | Kapplinger (2010) Heart Rhythm **7,** 33 |
| p.L917R | 102 | PRO | DAM | Yes | Yes | Kapplinger (2010) Heart Rhythm **7,** 33 |
| p.N927S | 46 | BEN | DAM | Yes | No | Meregalli (2006) J Cardiovasc Electrophysiol **17,** 857 |
| p.L928P | 98 | POS | DAM | Yes | Yes | Kapplinger (2010) Heart Rhythm **7,** 33 |
| p.L935P | 98 | PRO | DAM | Yes | Yes | Kapplinger (2010) Heart Rhythm **7,** 33 |
| p.R965C | 180 | PRO | DAM | Yes | Yes | Priori (2002) Circulation **105,** 1342 |
| p.R965H | 29 | PRO | DAM | Yes | Yes | Meregalli (2006) J Cardiovasc Electrophysiol **17,** 857 |
| p.A997T | 58 | BEN | TOL | No | No | Kapplinger (2010) Heart Rhythm **7,** 33 |
| p.R1023H | 29 | BEN | TOL | No | No | Frustaci (2005) Circulation **112,** 3680 |
| p.E1053K | 56 | PRO | DAM | Yes | Yes | Priori (2002) Circulation **105,** 1342 |
| p.D1055G | 94 | BEN | TOL | Yes | No | Kapplinger (2010) Heart Rhythm **7,** 33 |
| p.Q1059X | NA | NA | NA | NA | NA | Crotti (2012) J Am Coll Cardiol **epub,** epub |
| p.S1079Y | 144 | POS | DAM | Yes | Yes | Kapplinger (2010) Heart Rhythm **7,** 33 |
| p.A1113V | 64 | BEN | TOL | Yes | No | Kapplinger (2010) Heart Rhythm **7,** 33 |
| p.W1115X | NA | NA | NA | NA | NA | Kapplinger (2010) Heart Rhythm **7,** 33 |
| p.Q1118X | NA | NA | NA | NA | NA | Priori (2002) Circulation **105,** 1342 |
| p.S1140T | 58 | PRO | DAM | Yes | Yes | Kapplinger (2010) Heart Rhythm **7,** 33 |
| p.E1152X | NA | NA | NA | NA | NA | Garcia-Molina (2012) Clin Genet. 17 |
| p.W1191X | NA | NA | NA | NA | NA | Shin (2007) Life Sci **80,** 716 |
| p.W1192X | NA | NA | NA | NA | NA | Kapplinger (2010) Heart Rhythm **7,** 33 |
| p.S1218I | 142 | PRO | DAM | Yes | Yes | Calloe (2013) Circ Arrhythm Electrophysiol **6,** 1 |
| p.S1219N | 46 | PRO | DAM | Yes | Yes | Kapplinger (2010) Heart Rhythm **7,** 33 |
| p.E1225K | 56 | PRO | DAM | Yes | Yes | Smits (2002) J Am Coll Cardiol **40,** 350 |
| p.Y1228H | 83 | POS | TOL | Yes | No | Kapplinger (2010) Heart Rhythm **7,** 33 |
| p.R1232Q | 43 | PRO | DAM | Yes | Yes | Kapplinger (2010) Heart Rhythm **7,** 33 |
| p.K1236R | 26 | POS | TOL | Yes | No | Hermida (2010) Am J Cardiol **106,** 1758 |
| p.K1236N | 94 | PRO | DAM | Yes | Yes | Priori (2002) Circulation **105,** 1342 |
| p.L1239P | 98 | PRO | DAM | Yes | Yes | Kapplinger (2010) Heart Rhythm **7,** 33 |
| p.E1240Q | 29 | POS | DAM | Yes | Yes | Priori (2002) Circulation **105,** 1342 |
| p.D1243N | 43 | PRO | DAM | Yes | Yes | Kapplinger (2010) Heart Rhythm **7,** 33 |
| p.V1249D | 152 | POS | DAM | No | Yes | Kapplinger (2010) Heart Rhythm **7,** 33 |
| p.E1253G | 98 | PRO | DAM | Yes | Yes | Kapplinger (2010) Heart Rhythm **7,** 33 |
| p.G1262S | 56 | PRO | DAM | Yes | Yes | Shin (2004) J Hum Genet **49,** 573 |
| p.N1269S | 46 | BEN | TOL | No | No | Crotti (2012) J Am Coll Cardiol **epub,** epub |
| p.W1271C | 215 | PRO | DAM | Yes | Yes | Kapplinger (2010) Heart Rhythm **7,** 33 |
| p.V1281F | 50 | PRO | DAM | Yes | Yes | Hermida (2010) Am J Cardiol **106,** 1758 |
| p.A1288G | 60 | POS | DAM | Yes | Yes | Kapplinger (2010) Heart Rhythm **7,** 33 |
| p.F1293S | 155 | BEN | TOL | No | No | Priori (2002) Circulation **105,** 1342 |
| p.L1311P | 98 | PRO | DAM | Yes | Yes | Kapplinger (2010) Heart Rhythm **7,** 33 |
| p.G1319V | 109 | PRO | DAM | Yes | Yes | Smits (2002) J Am Coll Cardiol **40,** 350 |
| p.V1323G | 109 | PRO | DAM | Yes | Yes | Kapplinger (2010) Heart Rhythm **7,** 33 |
| p.V1340I | 29 | PRO | DAM | Yes | Yes | Kapplinger (2010) Heart Rhythm **7,** 33 |
| p.F1344L | 22 | PRO | DAM | Yes | Yes | Kapplinger (2010) Heart Rhythm **7,** 33 |
| p.F1344S | 155 | PRO | DAM | Yes | Yes | Keller (2006) Cardiovasc Res **70,** 521 |
| p.W1345C | 215 | PRO | DAM | Yes | Yes | Lee (2010) Korean Circ J **40,** 143 |
| p.L1346I | 5 | PRO | DAM | Yes | Yes | Kapplinger (2010) Heart Rhythm **7,** 33 |
| p.L1346P | 98 | PRO | DAM | Yes | Yes | Kapplinger (2010) Heart Rhythm **7,** 33 |
| p.I1350T | 89 | PRO | DAM | Yes | Yes | Juang (2003) Cardiology **99,** 182 |
| p.M1351R | 91 | PRO | DAM | Yes | Yes | Kapplinger (2010) Heart Rhythm **7,** 33 |
| p.V1353M | 21 | PRO | DAM | Yes | Yes | Kapplinger (2010) Heart Rhythm **7,** 33 |
| p.G1358W | 184 | PRO | DAM | Yes | Yes | Kapplinger (2010) Heart Rhythm **7,** 33 |
| p.K1359N | 94 | PRO | DAM | Yes | Yes | Kapplinger (2010) Heart Rhythm **7,** 33 |
| p.F1360C | 205 | PRO | DAM | Yes | Yes | Kapplinger (2010) Heart Rhythm **7,** 33 |
| p.C1363Y | 194 | PRO | DAM | Yes | Yes | Meregalli (2006) J Cardiovasc Electrophysiol **17,** 857 |
| p.L1373X | NA | NA | NA | NA | NA | Kapplinger (2010) Heart Rhythm **7,** 33 |
| p.V1378M | 21 | PRO | DAM | No | No | Moreau (2012) Front Pharmacol **3,** 62 |
| p.N1380K | 94 | PRO | DAM | Yes | Yes | Crotti (2008) Hum Genet **123** 542 |
| p.S1382I | 142 | PRO | DAM | Yes | Yes | Smits (2002) J Am Coll Cardiol **40,** 350 |
| p.Q1383X | NA | NA | NA | NA | NA | Kapplinger (2010) Heart Rhythm **7,** 33 |
| p.Y1394X | NA | NA | NA | NA | NA | Kapplinger (2010) Heart Rhythm **7,** 33 |
| p.V1405L | 32 | PRO | DAM | Yes | Yes | Smits (2002) J Am Coll Cardiol **40,** 350 |
| p.V1405M | 21 | PRO | DAM | Yes | Yes | Kapplinger (2010) Heart Rhythm **7,** 33 |
| p.G1406R | 125 | PRO | DAM | Yes | Yes | Smits (2002) J Am Coll Cardiol **40,** 350 |
| p.G1406E | 98 | PRO | DAM | Yes | Yes | Kapplinger (2010) Heart Rhythm **7,** 33 |
| p.G1408R | 125 | PRO | DAM | Yes | Yes | Kyndt (2001) Circulation **104,** 3081 |
| p.Y1409C | 194 | PRO | DAM | Yes | Yes | Kapplinger (2010) Heart Rhythm **7,** 33 |
| p.Y1409X | NA | NA | NA | NA | NA | Kapplinger (2010) Heart Rhythm **7,** 33 |
| p.L1412F | 22 | PRO | DAM | Yes | Yes | Kapplinger (2010) Heart Rhythm **7,** 33 |
| p.K1419E | 56 | PRO | DAM | Yes | Yes | Kapplinger (2010) Heart Rhythm **7,** 33 |
| p.G1420R | 125 | PRO | DAM | Yes | Yes | Kapplinger (2010) Heart Rhythm **7,** 33 |
| p.G1420V | 109 | PRO | DAM | Yes | Yes | Hermida (2010) Am J Cardiol **106,** 1758 |
| p.A1427S | 99 | POS | DAM | Yes | Yes | Kapplinger (2010) Heart Rhythm **7,** 33 |
| p.A1428V | 64 | PRO | DAM | Yes | Yes | Kapplinger (2010) Heart Rhythm **7,** 33 |
| p.R1432G | 125 | POS | DAM | Yes | Yes | Deschenes (2000) Cardiovasc Res **46,** 55 |
| p.G1433V | 109 | PRO | DAM | No | Yes | Kapplinger (2010) Heart Rhythm **7,** 33 |
| p.Y1434X | NA | NA | NA | NA | NA | Kapplinger (2010) Heart Rhythm **7,** 33 |
| p.P1438L | 98 | PRO | DAM | Yes | Yes | Six (2008) Europace **10,** 79 |
| p.W1440X | NA | NA | NA | NA | NA | Kapplinger (2010) Heart Rhythm **7,** 33 |
| p.E1441Q | 29 | PRO | DAM | Yes | Yes | Kapplinger (2010) Heart Rhythm **7,** 33 |
| p.N1443S | 65 | POS | TOL | Yes | No | Garcia-Molina (2012) Clin Genet. 17 |
| p.I1448L | 5 | BEN | TOL | Yes | No | Kapplinger (2010) Heart Rhythm **7,** 33 |
| p.I1448T | 89 | POS | DAM | Yes | Yes | Kapplinger (2010) Heart Rhythm **7,** 33 |
| p.Y1449C | 194 | PRO | DAM | Yes | Yes | Kapplinger (2010) Heart Rhythm **7,** 33 |
| p.V1451D | 152 | PRO | DAM | Yes | Yes | Kapplinger (2010) Heart Rhythm **7,** 33 |
| p.N1463Y | 143 | POS | DAM | Yes | Yes | Kapplinger (2010) Heart Rhythm **7,** 33 |
| p.V1468F | 50 | PRO | DAM | Yes | Yes | Kapplinger (2010) Heart Rhythm **7,** 33 |
| p.Q1476X | NA | NA | NA | NA | NA | Kapplinger (2010) Heart Rhythm **7,** 33 |
| p.K1493X | NA | NA | NA | NA | NA | Kapplinger (2010) Heart Rhythm **7,** 33 |
| p.Y1494N | 143 | POS | DAM | Yes | Yes | Tian (2007) Zhonghua Xin Xue Guan Bing Za Zhi **35,**1122 |
| p.G1502S | 56 | PRO | DAM | Yes | Yes | Smits (2002) J Am Coll Cardiol **40,** 350 |
| p.R1512W | 101 | PRO | DAM | Yes | Yes | Rook (1999) Cardiovasc Res **44,** 507 |
| p.I1521K | 102 | BEN | DAM | No | No | Kapplinger (2010) Heart Rhythm **7,** 33 |
| p.V1525M | 21 | POS | DAM | Yes | Yes | Kapplinger (2010) Heart Rhythm **7,** 33 |
| p.K1527R | 26 | BEN | TOL | Yes | No | Yokoi (2005) Heart Rhythm **3,** 2 |
| p.E1548K | 56 | POS | DAM | Yes | Yes | Kapplinger (2010) Heart Rhythm **7,** 33 |
| p.S1553R | 110 | BEN | DAM | Yes | Yes | Nakajima (2011) Int Heart J **52,** 27 |
| p.A1569P | 27 | POS | TOL | Yes | No | Yokoi (2005) Heart Rhythm **3,** 2 |
| p.F1571C | 205 | PRO | DAM | Yes | Yes | Kapplinger (2010) Heart Rhythm **7,** 33 |
| p.E1574K | 56 | PRO | DAM | Yes | Yes | Zumhagen (2009) Circ Arrhythm Electrophysiol **2,** 16 |
| p.L1582P | 98 | PRO | DAM | Yes | Yes | Amin (2009) Circ Arrhythm Electrophysiol **2,** 531 |
| p.R1583C | 180 | PRO | DAM | Yes | Yes | Kapplinger (2010) Heart Rhythm **7,** 33 |
| p.R1583H | 29 | PRO | DAM | Yes | Yes | Kapplinger (2010) Heart Rhythm **7,** 33 |
| p.W1591X | NA | NA | NA | NA | NA | Kapplinger (2010) Heart Rhythm **7,** 33 |
| p.V1604M | 21 | POS | DAM | Yes | Yes | Kapplinger (2010) Heart Rhythm **7,** 33 |
| p.Q1613L | 113 | BEN | DAM | Yes | Yes | Kapplinger (2010) Heart Rhythm **7,** 33 |
| p.Y1615X | NA | NA | NA | NA | NA | Kapplinger (2010) Heart Rhythm **7,** 33 |
| p.R1629Q | 43 | PRO | DAM | Yes | Yes | Kapplinger (2010) Heart Rhythm **7,** 33 |
| p.R1629G | 125 | PRO | DAM | Yes | Yes | Amin (2009) Circ Arrhythm Electrophysiol **2,** 531 |
| p.R1629X | NA | NA | NA | NA | NA | Kapplinger (2010) Heart Rhythm **7,** 33 |
| p.R1638X | NA | NA | NA | NA | NA | Meregalli (2006) J Cardiovasc Electrophysiol **17,** 857 |
| p.G1642E | 98 | PRO | DAM | Yes | Yes | Kapplinger (2010) Heart Rhythm **7,** 33 |
| p.R1644C | 180 | PRO | DAM | Yes | Yes | Frustaci (2005) Circulation **112,** 3680 |
| p.A1649V | 64 | PRO | DAM | Yes | Yes | Liang (2006) Zhonghua Xin Xue Guan Bing Za Zhi **34,** 616 |
| p.G1661R | 125 | PRO | DAM | Yes | Yes | Kapplinger (2010) Heart Rhythm **7,** 33 |
| p.S1672Y | 144 | PRO | DAM | No | Yes | Kapplinger (2010) Heart Rhythm **7,** 33 |
| p.D1690N | 23 | PRO | DAM | Yes | Yes | Núnez (2013) Heart Rhythm **10,** 2 |
| p.A1698T | 58 | PRO | DAM | Yes | Yes | Kapplinger (2010) Heart Rhythm **7,** 33 |
| p.Q1706H | 24 | PRO | DAM | Yes | Yes | Nakajima (2011) Int Heart J **52,** 27 |
| p.T1709R | 71 | PRO | DAM | Yes | Yes | Kapplinger (2010) Heart Rhythm **7,** 33 |
| p.T1709M | 81 | PRO | DAM | Yes | Yes | Yokokawa (2007) Am J Cardiol **100,** 649 |
| p.G1712S | 56 | PRO | DAM | Yes | Yes | Kapplinger (2010) Heart Rhythm **7,** 33 |
| p.D1714G | 94 | PRO | DAM | Yes | Yes | Amin (2005) Acta Physiol Scand **185,** 291 |
| p.L1717P | 98 | PRO | DAM | Yes | Yes | Crotti (2012) J Am Coll Cardiol **epub,** epub |
| p.N1722D | 23 | PRO | TOL | Yes | No | Probst (2009) Circ Cardiovasc Genet **2,** 552 |
| p.C1728R | 180 | PRO | DAM | Yes | Yes | Kapplinger (2010) Heart Rhythm **7,** 33 |
| p.C1728W | 215 | PRO | DAM | Yes | Yes | Kapplinger (2010) Heart Rhythm **7,** 33 |
| p.G1740R | 125 | PRO | DAM | Yes | Yes | Priori (2002) Circulation **105,** 1342 |
| p.G1743R | 125 | PRO | DAM | Yes | Yes | Takahata (2003) Life Sci **72,** 2391 |
| p.G1743E | 98 | PRO | DAM | Yes | Yes | Smits (2002) J Am Coll Cardiol **40,** 350 |
| p.G1748D | 94 | PRO | DAM | Yes | Yes | Núnez (2013) Heart Rhythm **10,** 2 |
| p.V1764F | 50 | PRO | DAM | Yes | Yes | Zumhagen (2009) Circ Arrhythm Electrophysiol **2,** 16 |
| p.N1774S | 46 | PRO | DAM | Yes | Yes | Du (2005) Zhongguo Yi Xue Ke Xue Yuan Xue Bao **27,**289 |
| p.L1786Q | 113 | PRO | DAM | Yes | Yes | Hofman-Bang (2006) Clin Genet **69,** 504 |
| p.Y1795H | 83 | PRO | DAM | Yes | Yes | Rivolta (2001) J Biol Chem **276,** 30623 |
| p.S1812X | NA | NA | NA | NA | NA | Schulze-Bahr (2003) Hum Mutat **21,** 651 |
| p.Q1832E | 29 | BEN | DAM | No | No | Arbustini (2005) Hum Genet **118** 536 |
| p.C1850S | 112 | POS | DAM | Yes | Yes | Petitprez (2008) Cardiovasc Res **78,** 494 |
| p.V1861I | 29 | POS | DAM | Yes | Yes | Kapplinger (2010) Heart Rhythm **7,** 33 |
| p.K1872N | 94 | PRO | DAM | Yes | Yes | Kapplinger (2010) Heart Rhythm **7,** 33 |
| p.A1924T | 58 | BEN | DAM | Yes | No | Rook (1999) Cardiovasc Res **44,** 507 |
| p.G1935S | 56 | BEN | TOL | Yes | Yes | Coronel (2005) Circulation **112,** 2769 |
| p.E1938K | 56 | BEN | TOL | No | No | Kapplinger (2010) Heart Rhythm **7,** 33 |
| p.A1949P | 27 | POS | DAM | Yes | Yes | Kotta (2010) Int J Cardiol **145,** 45 |
| p.V1951L | 32 | BEN | TOL | No | No | Priori (2002) Circulation **105,** 1342 |
| p.S1964F | 155 | POS | DAM | Yes | Yes | Hermida (2010) Am J Cardiol **106,** 1758 |
| p.I1968S | 142 | BEN | TOL | No | No | Frustaci (2005) Circulation **112,** 3680 |
| p.F2004L | 22 | BEN | TOL | No | No | Bebarova (2008) Am J Physiol Heart Circ Physiol **295,** H48 |

POS: Possibly damaging. PRO: Probably damaging. BEN: Benign. DAM: Damaging. TOL: Tolerated. CAS: Conservation across species. NA: Not available but nonsense mutations are likely disease causing.

**Supplementary table S2. *SCN5A* splicing mutations associated with BrS.**

| **Nucleotide** | **Reference** |
| --- | --- |
| c.274-24C>T | Rook (1999) Cardiovasc Res 44, 507 |
| c.393-1C>T | Nakajima (2011) Int Heart J 52, 27 |
| c.612-2A>G | Probst (2009) Circ Cardiovasc Genet 2, 552 |
| c.934+1G>A | Amin (2009) Circ Arrhythm Electrophysiol 2, 531 |
| c.934+4C>T | Kapplinger (2010) Heart Rhythm 7, 33 |
| c.998+1G>A | Kapplinger (2010) Heart Rhythm 7, 33 |
| c.1338+2T>A | Kapplinger (2010) Heart Rhythm 7, 33 |
| c.1890G>A | Kapplinger (2010) Heart Rhythm 7, 33 |
| c.1890+5G>A | Kapplinger (2010) Heart Rhythm 7, 33 |
| c.2024-1G>C | Priori (2002) Circulation 105, 1342 |
| c.3391-1G>A | García-Castro (2010) Rev Esp Cardiol 63, 856 |
| c.3666+1G>A | Smits (2002) J Am Coll Cardiol 40, 350 |
| c.3840+1G>A | Amin (2009) Circ Arrhythm Electrophysiol 2, 531 |
| c.3963+4A>G | Kapplinger (2010) Heart Rhythm 7, 33 |
| c. Not yet available | Rook (1999) Cardiovasc Res 44, 507 |
| c.4299G>A | Kapplinger (2010) Heart Rhythm 7, 33 |
| c.4299+1G>T | Kapplinger (2010) Heart Rhythm 7, 33 |
| c.4300-1G>A | Kapplinger (2010) Heart Rhythm 7, 33 |
| c.4438-1C>T | Shimada (2012) Int J Cardiol epub, epub |
| c.4719C>T | Amin (2009) Circ Arrhythm Electrophysiol 2, 531 |

**Supplementary table S3. *SCN5A* deletions associated with BrS.**

| **Nucleotide** | **Reference** |
| --- | --- |
| c.191_193delTGC | Kapplinger (2010) Heart Rhythm 7, 33 |
| c.486delC | Kapplinger (2010) Heart Rhythm 7, 33 |
| c.483-10_492del20 | Vatta (2002) Mol Genet Metab 75, 317 |
| c.1177_1179delTTC | Priori (2002) Circulation 105, 1342 |
| c.1428_1431delCAAG | Kapplinger (2010) Heart Rhythm 7, 33 |
| c.1537delC | Kapplinger (2010) Heart Rhythm 7, 33 |
| c.1562delA | Kapplinger (2010) Heart Rhythm 7, 33 |
| c.1721delG | Kapplinger (2010) Heart Rhythm 7, 33 |
| c.1936delC | Kapplinger (2010) Heart Rhythm 7, 33 |
| c.1950_1953delAGAT | Kapplinger (2010) Heart Rhythm 7, 33 |
| c.2024_2025delAG | Kapplinger (2010) Heart Rhythm 7, 33 |
| c.2274delG | Kapplinger (2010) Heart Rhythm 7, 33 |
| c.2327_2329delACT | Kapplinger (2010) Heart Rhythm 7, 33 |
| c.2533delG | Kapplinger (2010) Heart Rhythm 7, 33 |
| c.2541delC | Hsueh (2009) J Biomed Sci 16, 23 |
| c.2545_2547delATC | Bokeria (2007) Vestn Ross Akad Med Nauk , 3 |
| c.2582_2583delTT | Schulze-Bahr (2003) Hum Mutat 21, 651 |
| c.2602delC | Schulze-Bahr (2003) Hum Mutat 21, 651 |
| c.2613delC | Priori (2002) Circulation 105, 1342 |
| c.2799_2800delCT | Hermida (2010) Am J Cardiol 106, 1758 |
| c.2850delT | Kapplinger (2010) Heart Rhythm 7, 33 |
| c.2914_2923del10 | Kapplinger (2010) Heart Rhythm 7, 33 |
| c.3005_3012delCCAGCTGC | Zumhagen (2009) Circ Arrhythm Electrophysiol 2, 16 |
| c.3045_3046delGA | Hedley (2009) Hum Mutat 30, 1256 |
| c.3228+2delT | Kapplinger (2010) Heart Rhythm 7, 33 |
| c.3480delT | Hofman-Bang (2006) Clin Genet 69, 504 |
| c.3553_3554delCA | Kapplinger (2010) Heart Rhythm 7, 33 |
| c.3666+1delG | Kapplinger (2010) Heart Rhythm 7, 33 |
| c.3667delG | Probst (2009) Circ Cardiovasc Genet 2, 552 |
| c.3894delC | Kapplinger (2010) Heart Rhythm 7, 33 |
| c.3940_3941delCT | Millat (2009) Clin Biochem 42, 491 |
| c.4067_4068delTT | Möbius-Winkler (2011) Heart Rhythm epub, epub |
| c.4376_4379delTCTT | Kapplinger (2010) Heart Rhythm 7, 33 |
| c.4389_4396delCCTCTTTA | Kapplinger (2010) Heart Rhythm 7, 33 |
| c.4402_4405delGTCA | Priori (2002) Circulation 105, 1342 |
| c.4436_4437+1delAGA | Smits (2002) J Am Coll Cardiol 40, 350 |
| c.4477_4479delAAG | Zumhagen (2009) Circ Arrhythm Electrophysiol 2, 16 |
| c.4856delC | Kapplinger (2010) Heart Rhythm 7, 33 |
| c.5068_5069delGA | Kapplinger (2010) Heart Rhythm 7, 33 |
| c.5124_5126delCAC | Kapplinger (2010) Heart Rhythm 7, 33 |
| c.5157delC | Yokokawa (2007) Am J Cardiol 100, 649 |
| c.5290delG | Yokokawa (2007) Am J Cardiol 100, 649 |
| incl. ex. 9-10 | Eastaugh (2011) J Cardiovasc Electrophysiol **22,** 1073 |
| c.5324delT | Baroudi (2004) Can J Cardiol 20, 425 |
| c.5356_5357delCT | Kapplinger (2010) Heart Rhythm 7, 33 |

**Supplementary table S4. *SCN5A* small insertions associated with BrS.**

| **Nucleotide** | **Reference** |
| --- | --- |
| c.381dupT | Kapplinger (2010) Heart Rhythm 7, 33 |
| c.410_418dupTCATGTGCA | Kapplinger (2010) Heart Rhythm 7, 33 |
| c.656_657insATTCA | Kapplinger (2010) Heart Rhythm 7, 33 |
| c.2201dupT | Kapplinger (2010) Heart Rhythm 7, 33 |
| c.2549_2550insTG | Kapplinger (2010) Heart Rhythm 7, 33 |
| c.2953dupC | Krahn (2009) Circulation 120, 278 |
| c.4299+1dupG | Skinner (2007) Pediatrics 119, e1206 |
| c.4708_4710dupATC | Tan (2005) Circulation 112, 207 |
| c.4732_4733dupAA | Makiyama (2005) J Am Coll Cardiol 46, 2100 |
| c.4813+3_4813+6dupGGGT | Hong (2005) J Mol Cell Cardiol 38, 555 |
| c.4952dupT | Crotti (2012) J Am Coll Cardiol epub, epub |
| c.5578dupA | Kapplinger (2010) Heart Rhythm 7, 33 |
| c.5623_5625dupATG | Hsueh (2009) J Biomed Sci 16, 23 |
| c.6010_6012dupTTC | Kapplinger (2010) Heart Rhythm 7, 33 |

**Supplementary table S5. *SCN5A* small indels associated with BrS.**

| **Nucleotide** | **Reference** |
| --- | --- |
| 2435_2436+3delTGGTAinsCGCCT | Kapplinger (2010) Heart Rhythm 7, 33 |
| 3171_3172delTGinsA | Kapplinger (2010) Heart Rhythm 7, 33 |
| 5040_5042delTTAinsC | Kranjcec (2007) Pacing Clin Electrophysiol 30, 1294 |
